# Organic/Inorganic Nano-hybrids with High Dielectric Constant for Organic Thin Film Transistor Applications

**DOI:** 10.1186/s11671-016-1710-4

**Published:** 2016-11-07

**Authors:** Yang-Yen Yu, Ai-Hua Jiang, Wen-Ya Lee

**Affiliations:** 1 Department of Materials Engineering, Ming Chi University of Technology, No. 84, Gongzhuan Rd., Taishan Dist., New Taipei City, 24301 Taiwan; 2 Department of Chemical and Materials Engineering, Chang Gung University, No.259, Wenhua 1st Rd., Guishan Dist., Taoyuan, 33302 Taiwan; 3 Department of Chemical Engineering and Biotechnology, National Taipei University of Technology, No 43, Keelung Rd., Sec.4, Da’an Dist., Taipei, 10607 Taiwan

**Keywords:** Polyimide, Barium titanate, Hybrid films, Sol–gel method, Modified layer

## Abstract

The organic material soluble polyimide (PI) and organic–inorganic hybrid PI–barium titanate (BaTiO_3_) nanoparticle dielectric materials (IBX, where X is the concentration of BaTiO_3_ nanoparticles in a PI matrix) were successfully synthesized through a sol–gel process. The effects of various BaTiO_3_ contents on the hybrid film performance and performance optimization were investigated. Furthermore, pentacene-based organic thin film transistors (OTFTs) with PI-BaTiO_3_/polymethylmethacrylate or cyclic olefin copolymer (COC)-modified gate dielectrics were fabricated and examined. The hybrid materials showed effective dispersion of BaTiO_3_ nanoparticles in the PI matrix and favorable thermal properties. X-ray diffraction patterns revealed that the BaTiO_3_ nanoparticles had a perovskite structure. The hybrid films exhibited high formability and planarity. The IBX hybrid dielectric films exhibited tunable insulating properties such as the dielectric constant value and capacitance in ranges of 4.0–8.6 and 9.2–17.5 nF cm^−2^, respectively. Adding the modified layer caused the decrease of dielectric constant values and capacitances. The modified dielectric layer without cross-linking displayed a hydrophobic surface. The electrical characteristics of the pentacene-based OTFTs were enhanced after the surface modification. The optimal condition for the dielectric layer was 10 wt% hybrid film with the COC-modified layer; moreover, the device exhibited a threshold voltage of 0.12 V, field-effect mobility of 4.32 × 10^−1^ cm^2^ V^−1^ s^−1^, and on/off current of 8.4 × 10^7^.

## Background

Organic thin film transistors (OTFTs) have attracted considerable attention in recent years for their unique features, including low fabrication costs [1–5], flexibility [6, 7], and ease of processing in solution [8]. Relevant research has sought to improve OTFT performance in organic and polymeric semiconductors by modifying their chemical structures [9–12]. Other approaches, such as controlling the deposition of crystalline organic films [13–15] and controlling the nature of the interfaces, have also been developed [16, 17].

In OTFTs, improving the semiconductor/dielectric interface has involved modifying the gate dielectrics with polymeric materials, organic–inorganic hybrids, and an organic–inorganic bilayer [18, 19]. Organic–inorganic hybrid material is a new type of material that demonstrates the desirable physical properties of both organic and inorganic components within a single composite. Moreover, inorganic material, such as a metal oxide, has a high-dielectric-constant material [20, 21]. These hybrid materials can enhance gate capacitance for accumulating more charge carriers in the channel. Additionally, the dielectric properties of mixtures of polymers and inorganic nanofillers [22, 23] including nanoparticles [24, 25], nanoclusters, and nanotubes [26, 27] can be tuned by varying the type and concentration of nanofiller materials.

In the literature, many polymeric dielectric materials have been applied as the dielectric materials in OTFTs, such as poly(styrene), poly(methyl methacrylate), poly(ethylene), poly(urethane), poly(vinyl alcohol), and poly(vinyl pyridine). However, among these polymeric dielectrics, polyimide (PI) is the best one and has been widely used as an insulating material for application in the field of electronic components due to its lower leakage current density, good thermal stability, mechanical toughness, and chemical resistance. In addition, the soluble polyimide can be applied to a low-temperature process and thus can prevent the need of high-temperature reaction for the dehydration and cyclization. On the other hand, barium titanate (BaTiO_3_) nanoparticles have a large dielectric constant and behave similarly to ferroelectric dielectric materials. Polyimide–BaTiO_3_ hybrid thin films providing high-quality dielectric nanocomposite materials were produced using simple solution techniques. Using the hybrid materials of high-dielectric-constant BaTiO_3_ nanoparticles in the PI matrix as gate dielectric materials can improve the performance of OTFT devices. In particular, to improve the electric characteristics and operational stability of OTFTs, the gate dielectric layers were modified with hydroxyl-free polymer insulators such as polymethylmethacrylate (PMMA) and cyclic olefin copolymer (COC).

## Methods

### Materials

4,4′-(Hexafluoroisopropylidene)dianiline (Lenexa, USA, 99 %), 4,4′-(hexafluoroisopropylidene)diphthalic anhydride (Alfa Aesar, 98 %), and 4-aminobenzoic acid (ACROS, 99.5 %) were used to synthesize PI. Then, the prepared PI and barium titanate (Seechem Company PTYLTD, 99 %) was used as inorganic nanoparticles to prepare the hybrid dielectric films (PI-BaTiO_3_, IBX), and COC (Polyscience Inc.) and PMMA (Alfa Aesar, 98 %) were used as the polymer dielectric part. There are two kinds of solvents, tetrahydrofuran (ACROS, 99.9 %) and *N*,*N*-dimethylacetamide (ACROS, 99.8 %). Pentacene, an organic semiconductor material, was purchased from TCI Co. Ltd.

### Synthesis of PI–BaTiO_3_ Hybrid Films

A solution–imidization technique was utilized to synthesize organo-soluble polyimide (6FDA–6FpDA–COOH) with carboxylic acid end groups [28]. The molecular weight and end group functionality were controlled by the reactant stoichiometry. Firstly, 2.01 g (0.006 mol) of 4,4^′^-(hexafluoroisopropylidene)dianiline (6FpDA) was added into a 100-ml three-necked round-bottom flask, and 29.1 ml of NMP was used to dissolve the reactants. 5.331 g (0.012 mol) of 4,4^′^-(hexafluoroisopropylidene)diphthalic anhydride (6FDA) was then slowly added into the above solution with vigorous stirring under nitrogen purging. The mixture was allowed to react for 8 h at room temperature. Secondly, 1.6457 g (0.012 mol) of 4-aminobenzoic acid (4ABA) and 7.2 ml of 1,3-dichlorobenzene were added to the above solution. The 20 wt% of poly(amic acid) (PAA) solution was thus formed after constantly stirring the reactants for 16 h at room temperature. The PAA solution was then thermally imidized in a 1800 °C silicon oil bath for another 8 h and cooled to room temperature. The homogeneous 6FDA–6FpDA–4ABA–COOH solution was precipitated with 500 ml of methanol and re-dissolved in 30 ml of THF twice. A white-gray precipitate was recovered and dried in a vacuum oven at 1500 °C for 24 h to obtain 2.136 g of 6FDA–6FpDA–4ABA–COOH (yield, 23.8 %). The average acid value of 6FDA–6FpDA–4ABA–COOH was found to be 14 mg KOH/0.5 g polyimide using titration. The average molecular weight estimated by the acid value was around 4000. The weight average molecular weight estimated by GPC was 4276 with a polydispersity index of 1.31. It is noted that the yield of 6FDA–6FpDA–4ABA–COOH could be improved to nearly 50 % if monomers 6FDA, 6FpDA, and 4ABA were purified at 244–247, 195–198, and 187–189 °C, respectively, by sublimation/condensation procedure before the polyimide synthesis. The 6FDA–6FpDA–4ABA–COOH film was prepared using the following procedure: 0.5 g of 6FDA–6FpDA–4ABA–COOH was dissolved in 5 ml of DMAc while being stirred. The solution was filtered with a 0.45-m PTFE filter prior to use and spin-coated on to a silicon wafer at 1000 rpm for 20 s. The film was then baked at 60 °C on a hot plate for 10 min and at 150 °C for another 30 min to evaporate the solvent. The characteristic peaks of the FTIR spectrum for IB0 were observed as follows: 3434 cm^−1^ (COOH), 1788 cm^−1^ (CO), 1726 cm^−1^ (CO), 1610 cm^−1^ (C_6_H_5_), 1517 cm^−1^ (C_6_H_5_), 1438 cm^−1^ (C_6_H_5_), and 1370 cm^−1^ (CN) [28]. Next, the IBX hybrid solutions were prepared. The PI was mixed with different weight ratios of BaTiO_3_ (0, 2, 5, 8, 10, and 12 wt%, i.e., IB0–IB12) in DMAc solvent and stirred uniformly to form the IBX hybrid solution. To prepare the IBX hybrid thin films, the precursor solution was spin-coated onto a silicon substrate. Finally, the hybrid dielectric thin films were baked at 60 °C for 30 min, 100 °C for 30 min, and 150 °C for 60 min [28–30].

### Preparation of Modified Layer

Bilayer dielectrics consist of IBX hybrid films and a polymer layer. The dilute PMMA or COC was a mixture of monochlorobenzene and 1 % PMMA or COC. The volume ratio of monochlorobenzene and 1 % PMMA or COC was three to one. The bilayer dielectrics were baked at a temperature of 110 °C to remove moisture after being spin-coated onto the IBX thin films.

### Characterization of Prepared Hybrid Composites

The structure of the prepared IBX hybrid thin films was determined using Fourier transform infrared spectroscopy (Perkin–Elmer Spectrum One), Raman spectroscopy (HORIBA iHR550), and an X-ray diffractometer (PANalytical X’Pert PRO MPD) by using CuKα radiation. The thermal properties of the IBX hybrid materials were measured using a TA Instruments Thermogravimetric Analyzer (Mettler Toledo TGA/SDTA851) and a differential scanning calorimeter (Perkin–Elmer Pyris 1) with heating rates of 20 and 10 °C min^−1^. The transmittances of the hybrid films coated onto quartz substrates were analyzed using ultraviolet–visible spectroscopy (Jasco V650). The surface morphologies of the thin films were examined using ultrahigh-resolution field emission scanning electron microscopy (FE-SEM, JEOL JSM-6500) and a student module of atomic force microscopy (AFM, Veeco DI3100).

### OTFT Fabrication and Characterization

**Fig. 1:**
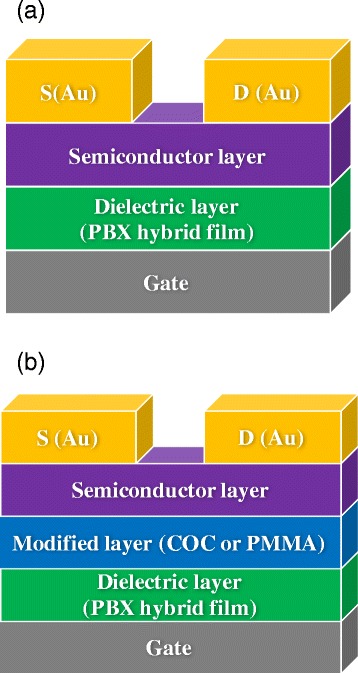
The schematic of the cross-section of the fabricated OTFTs: (**a**) without and (**b**) with a modified layer

Organic transistors were fabricated on Si substrates by using a bottom-gate top-contact (BGTC) structure. First, hybrid thin films and polymer dielectrics were spin-coated onto heavily doped n^+^ Si substrates as dielectric layers and then annealed. A 50-nm-thick layer of pentacene as a p-type semiconductor layer was deposited onto the dielectric layers through thermal evaporation deposition at a pressure of approximately 10^7^ Torr and a deposition rate of 0.5 Å s^−1^. Finally, a 50-nm-thick layer of gold was deposited onto the semiconductor layer through vacuum thermal evaporation and patterned through a shadow mask forming the source and drain electrodes. All devices had a channel length of 50 μm and a channel width of 1000 μm. To form the metal–insulator–metal (MIM) structures, a 100-nm-thick aluminum film used as an electrode was deposited directly onto the gate hybrid dielectric/Si films with a mask. The dielectric properties of the samples were characterized using a capacitance–voltage measurement system (Agilent B1500A). Frequency sweeps were performed from 10 kHz to 1 MHz. The current–voltage characteristics of the OTFTs were obtained at room temperature in air by using a semiconductor characterization system (Keithley 4200-SCS). Figure 1 shows the schematic of the cross-section of the fabricated OTFTs.

## Results and Discussion

The IBX hybrid materials were fabricated as the gate dielectrics of the OTFT devices. PI is a highly thermal and environmentally stable material; therefore, it is well suited for use as the dielectric matrix. PI was synthesized in a two-step polymerization process that included PAA synthesis and chemical imidization. Hybrid thin films were spin-coated using a precursor solution, followed by thermal curing, as described in the experimental section. The dispersion and aggregation behaviors of the nanoparticles exert a crucial effect on the properties of IBX dielectric materials.

### Structure Analysis of IBX Hybrid Dielectric Films

**Fig. 2:**
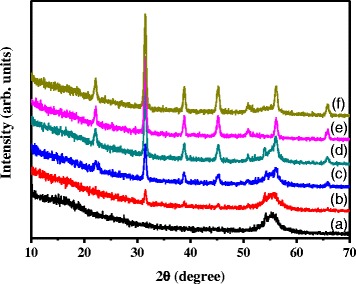
The XRD pattern of hybrid thin films for (*a*) IB0, (*b*) IB2, (*c*) IB5, (*d*) IB8, (*e*) IB10, and (*f*) IB12

The crystalline structures of the prepared IBX hybrids were analyzed using X-ray diffraction, as depicted in Fig. 2. The sharp X-ray reflections at 2*θ* = 22.0°, 31.5°, 38.7°, 45.1°, 50.8°, 56.1°, and 65.8° were assigned to (100), (110), (111), (200), (210), (211), and (220), respectively [29]. No other diffraction peaks were present. These characterized peaks show that the formation of the inorganic nanoparticles is in the perovskite phase. This figure shows that the diffraction peaks of BaTiO_3_ increased with the increasing contents of BaTiO_3_ in hybrids.

### Optical Analysis of IBX Hybrid Dielectric Films

**Fig. 3:**
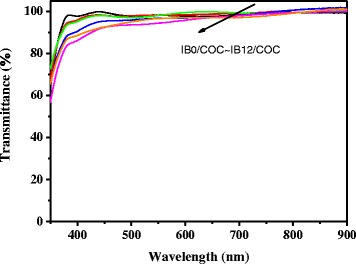
Optical transmittance spectra of IBX/COC hybrid thin films

Figure 3 shows optical transmittance spectra of the bilayer hybrid films on glass substrates over the entire visible region. The transmittances of the IBX–COC hybrid thin films are greater than 80 % in the measured region. Furthermore, as the content of the BaTiO_3_ nanoparticles increased in the composite films, the optical transparency decreased. The hybrid bilayer films, even at high BaTiO_3_ concentrations, exhibited favorable transmittance and were colorless. The superior transparency of the IBX–COC hybrid may result from the reduced formation of charge transfer complexes, and limited electronic conjugation along the PI backbone, that arise from the presence of fluorine moieties, the non-coplanar PI geometry, and the bulky IBX substituents present in PI the side chains. This property is beneficial for applications requiring transparency, such as in a high-resolution and brightness electronic display.

### Surface Analysis of IBX Hybrid Dielectric Films

**Fig. 4:**
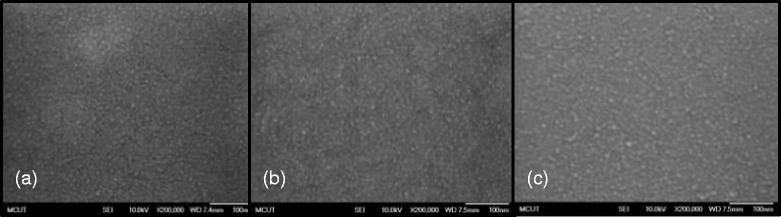
The FE-SEM images of dielectric surfaces for **a** IB10, **b** IB10/PMMA, and **c** IB10/COC

**Fig. 5:**
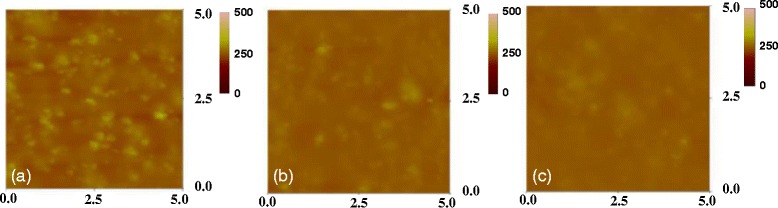
The AFM images of **a** IB10, **b** IB10/PMMA, and **c** IB10/COC dielectric surfaces

Figures 4 and 5 present the FE-SEM and AFM images of the IBX dielectric films at low temperature. In addition, the surface morphologies of (a) PB10, (b) PB10/PMMA, and (c) PB10/ COC bilayer thin films are also shown in these figures. The FE-SEM images of the hybrid surface demonstrate that the BaTiO_3_ nanoparticles were homogeneously dispersed in the PI matrix. Adding PMMA and COC flattened the surface of the dielectric films. The tapping mode AFM measurement provided the Ra roughnesses of the hybrid samples in the range of 0.319–9.007 nm (Table 1). These AFM images reveal that surface morphology is closely related to the BaTiO_3_ loading content in hybrid material. With the increasing BaTiO_3_ content of the PI matrix, the Ra values of the hybrid thin films increased. When the polymer was added to the IBX thin films, the Ra values decreased. Taking PB10 as an example, the Ra values of PB10, PB10–PMMA, and PB10–COC were measured at 8.324, 3.597, and 2.489 nm, respectively. Furthermore, the surfaces of all the hybrid dielectric samples were pinhole free. Overall, our observations suggest that IBX nanoparticles effectively disperse in the hybrid dielectric film PI matrix.

**Table 1:** Summary of roughness of hybrid thin films

Dielectric materials	No modified layer		PMMA-modified		COC-modified	
	Ra roughness (nm)	Thickness (nm)	Ra roughness (nm)	Thickness (nm)	Ra roughness (nm)	Thickness (nm)
IB0	0.319	352	0.262	375	0.233	381
IB2	1.836	358	1.301	377	1.159	380
IB5	3.761	363	2.484	387	1.535	379
IB8	5.390	354	3.187	370	2.189	374
IB10	8.324	365	3.597	389	2.489	383
IB12	9.007	356	4.340	373	3.229	380

The contact angles of the surfaces of the hybrid gate dielectric materials were measured. The surface energies of the hybrid gate dielectrics were quantified through calculation, based on the different polarities of deionized water and diiodomethane. Table 2 shows the contact angle and surface energy for the various hybrid dielectrics. A small water contact angle on the solid surface means that the surface is hydrophilic and possesses a large surface energy; conversely, a large contact angle indicates a hydrophobic surface with low surface energy. The PB0 films, without the addition of BaTiO_3_ nanoparticles, exhibited a small water contact angle of 68.7°. However, hybrid gate dielectrics incorporated with BaTiO_3_ nanoparticles showed increasing contact angles when the BaTiO_3_ content increased. The reason for this observation is that a greater BaTiO_3_ concentration presents fewer hydroxyl groups because of the condensation reaction between functional PI and the modified BaTiO_3_ nanoparticles. Hence, the surface energy decreased from 57.4 to 55.4 mJ m^2^ as the 10 wt.% BaTiO_3_ was added. The high-dielectric-constant BaTiO_3_ nanoparticles were surrounded by the low-dielectric-constant PI matrix, thus causing the surface energy to decrease. Moreover, the addition of hydroxyl-free polymer materials (PMMA or COC) yielded a more hydrophobic surface because of the polymer’s intrinsic hydrophobic property. In particular, the COC-modified dielectric surface presented a larger water contact angle than the others did, resulting in lower surface energy. The results showed that the lowest surface energy obtained from the PB10–COC hybrid material was 47.4 mJ m^2^ [31]. Generally, a dielectric surface with low surface energy provides sites for organic semiconductor chain growth.

**Table 2:** Summary of contact angle and surface energies data of hybrid thin films

Dielectric materials	No modified layer			PMMA-modified			COC-modified		
	Water contact angle	DIM contact angle	Surfaces energy (mJ m^−2^)	Water contact angle	DIM contact angle	Surfaces energy (mJ m^−2^)	Water contact angle	DIM contact angle	Surfaces energy (mJ m^−2^)
IB0	68.72°	28.71°	57.40	74.96°	27.49°	53.42	89.16°	28.16°	48.82
IB2	68.87°	32.22°	56.22	75.53°	28.95°	53.39	90.17°	28.41°	48.57
IB5	69.06°	33.16°	56.09	76.96°	29.35°	53.28	90.63°	30.50°	47.69
IB8	69.12°	33.25°	55.99	77.64°	31.98°	52.22	90.89°	31.05°	47.61
IB10	69.17°	34.41°	55.45	81.13°	36.09°	51.91	91.33°	32.54°	47.48
IB12	69.13°	33.41°	55.82	79.32°	34.51°	52.06	91.17°	31.06°	47.58

### OTFT Characteristics with IBX Hybrids as Dielectrics

**Fig. 6:**
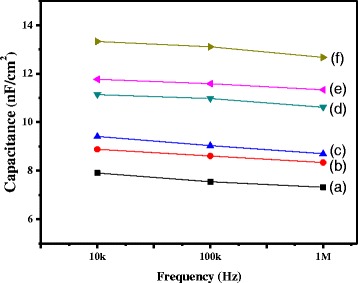
Capacitance vs. frequency plots for (*a*) IB0/COC, (*b*) IB2/COC, (*c*) IB5/COC, (*d*) IB8/COC, (*e*)IB10/COC, and (*f*) IB12/COC hybrid dielectrics

The dielectric properties of IBX films are strongly dependent on BaTiO_3_ content. The MIM capacitor device used for the capacitance measurements (Figs. 6) consisted of distinct IBX hybrid dielectric layers/bilayers sandwiched between two aluminum electrodes. Figure 6 shows the capacitance (*C*)–frequency (*F*) plots for a MIM structure prepared using IBX–COC dielectrics for different BaTiO_3_ loading. As the BaTiO_3_ content increased, the hybrid film’s capacitance increased. Adding BaTiO_3_ nanoparticles effectively improved the composites’ dielectric properties. The obtained hybrid capacitances were in the ranges of 9.2–17.5 nF cm^−2^ at 10 kHz for IBX, 8.4~14.7 nF cm^−2^ for IBX–PMMA, and 7.9~13.3 nF cm^−2^ for IBX–COC. Notably, in the range of 10 kHz^−1^ MHz, the BaTiO_3_ content exhibited a linear relationship with the frequency response. The capacitance increased slightly at a lower frequency perhaps because of the increase in the response time for polarization. The dielectric constants (*k*) can be calculated using the following function:

$$ C=\frac{k{\varepsilon}_0A}{d} $$

where C is the capacitance (in *F*), *ε*_0_ is the dielectric constant of vacuum, *A* is the area of the electrodes (in m^2^), and *d* is the thickness of the dielectric layer. The dielectric constants at 10 kHz are summarized in Table 3 [32]. The measured dielectric constant values of the hybrid films ranged 4.551–8.609 for IBX, 4.121–7.221 for IBX–PMMA, and 4.079~6.538 for IBX–COC. The dielectric constants of the hybrid materials can be increased by increasing the inorganic BaTiO_3_ nanoparticle content. This is because of the higher dielectric permittivity of the well-dispersed nanofiller, isolated by the passivating layers of the polymer matrix. The composite precursor solution undergoes a condensation reaction to form a –O–BaTiO_3_–O network interface resulting in the uniform incorporation of BaTiO_3_ nanoparticles in the PI phase. The various hybrid films were used for OTFT applications.

**Table 3:** Summary of electrical parameters for MIM device and pentacene OTFTs with different hybrid dielectrics

Dielectric materials	Capacitance (nF cm^−2^)	Dielectric constant (−)	μ (cm^2^ V^−1^ s^−1^)	*V*_t_ (V)	*I*_on_/*I*_off_ (−)
IB0	9.2	4.5	1.03 × 10^−1^	5.8	3.0 × 10^5^
IB2	10.3	5.0	1.86 × 10^−1^	−4.2	7.5 × 10^5^
IB5	11.2	5.5	2.38 × 10^−1^	5.3	1.2 × 10^6^
IB8	12.4	6.1	2.53 × 10^−1^	2.6	5.2 × 10^6^
IB10	16.0	7.8	2.76 × 10^−1^	−3.1	8.2 × 10^6^
IB12	17.5	8.6	2.57 × 10^−1^	−8.2	6.1 × 10^6^
IB0/PMMA	8.4	4.1	1.88 × 10^−1^	−1.8	7.1 × 10^5^
IB2/PMMA	9.2	4.5	2.72 × 10^−1^	0.3	1.3 × 10^6^
IB5/PMMA	10.1	4.9	2.97 × 10^−1^	4.2	2.7 × 10^6^
IB8/PMMA	12.0	5.9	3.64 × 10^−1^	4.3	9.4 × 10^6^
IB10/PMMA	12.6	6.2	4.17 × 10^−1^	2.7	2.2 × 10^7^
IB12/PMMA	14.7	7.2	3.81 × 10^−1^	2.9	1.5 × 10^7^
IB0/COC	7.9	4.0	2.01 × 10^−1^	−5.1	8.3 × 10^5^
IB2/COC	9.2	4.3	2.80 × 10^−1^	0.8	3.1 × 10^6^
IB5/COC	9.4	4.6	3.12 × 10^−1^	−1.6	5.1 × 10^6^
IB8/COC	11.1	5.6	3.81 × 10^−1^	−3.7	1.8 × 10^7^
IB10/COC	11.7	5.9	4.32 × 10^−1^	0.1	8.4 × 10^7^
IB12/COC	13.3	6.5	3.96 × 10^−1^	−2.6	2.0 × 10^7^

**Fig. 7:**
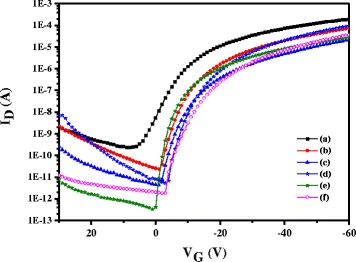
Transfer curves of OTFTs based on (*a*) IB0/COC, (*b*) IB2/COC, (*c*) IB5/COC, (*d*) IB8/COC, (*e*) IB10/COC, and (*f*) IB12/COC hybrid dielectrics

BGTC pentacene OTFTs with Au electrodes were fabricated with the IBX, IBX–PMMA, and IBX–COC hybrid films as the gate dielectrics. We determined the field-effect mobility (*μ*) from the transfer curves by using the following equation:

$$ {I}_{\mathrm{d}}=\frac{W{C}_{\mathrm{i}}}{2L}\mu {\left({V}_{\mathrm{g}}-{V}_{\mathrm{t}}\right)}^2 $$

where *I*_d_ is the drain current, *W*/*L* is the channel width to length ratio, *C*_i_ is the capacitance per unit area of the hybrid gate dielectric used, *V*_g_ is the gate voltage, and *V*_t_ is the threshold voltage. The field-effect mobility is calculated by plotting the square root of *I*_d_ versus *V*_g_. Figure 7 shows the transfer curves of the OTFTs with IBX–COC hybrid films as the gate dielectrics. All the electrical characteristics of these OTFTs are summarized in Table 3. Greater hole mobility is obtained for OTFTs with a larger BaTiO_3_ content. The PB10 dielectric with the mobility value of 2.76 × 10^−1^ cm^2^ V^−1^ s^−1^ provided the greatest performance in the IBX series. Relatively high OTFT on/off current ratios (*I*_on_/*I*_off_) over 10^5^ were observed for all the samples. Furthermore, the highest *I*_on_/*I*_off_ can be further improved to 10^7^ in the polymer-modified devices. In addition, all the threshold voltages were quite low. As observed, the addition of a polymer-modified layer significantly improved the electrical properties. This may be originated from the highly hydrophobic nature of the ethylene–norborene COC. The highest mobility of the OTFTs with PB10–COC hybrid dielectrics reached 4.32 × 10^−1^ cm^2^ V^−1^ s^−1^. According to these data, we concluded that the hybrid materials composed of the functional PI incorporated with 10 wt% BaTiO_3_ as the dielectric layer of OTFTs exhibited favorable electrical properties. Furthermore, introducing the polymer to modify the dielectrics produced high-performance p-type OTFTs.

**Fig. 8:**
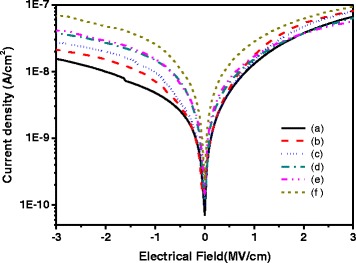
Capacitor leakage current curves of OTFTs based on (*a*)IB0/COC, (*b*) IB2/COC, (*c*) IB5/COC, (*d*) IB8/COC,(*e*)IB10/COC, and (*f*) IB12/COC hybrid dielectrics

Figure 8 shows the leakage characteristics of OTFTs based on (a) IB0/COC, (b) IB2/COC, (c) IB5/COC, (d) IB8/COC, (e) IB10/COC, and (f) IB12/COC hybrid dielectrics. It showed that the leakage current density increased with the increase in BaTiO_3_ content. The greater leakage current density at higher BaTiO_3_ content was caused by the larger capacitance and the surface roughness of hybrid dielectric layer. An increase in the leakage current means that the effect of the insulating layer is lowered. As shown in Fig. 8, the leakage current of all the dielectric layer was less than 10^−7^ A/cm at −2 MV/cm and demonstrated the resistant ability of current leakage for the hybrid dielectrics in this study. Therefore, these hybrid dielectric layers are suitable for organic thin film transistor applications. The gate current behavior is typically similar to capacitor leakage current. In this work, we used metal–insulator–metal capacitor to investigate dielectric leakage.

**Fig. 9:**
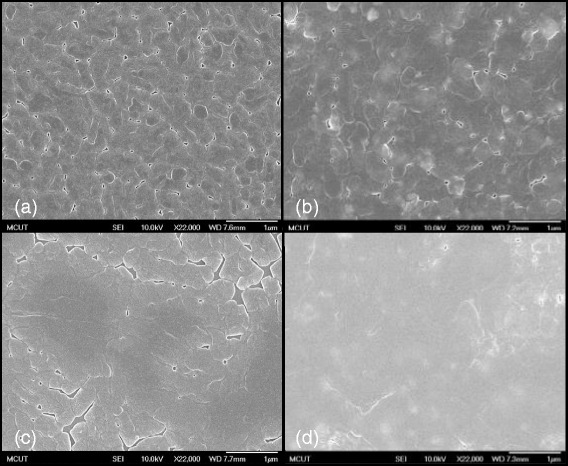
The FE-SEM images of pentacene deposited on **a** IB0, **b** IB10, **c** IB10/PMMA, and **d** IB10/COC dielectrics

**Fig. 10:**
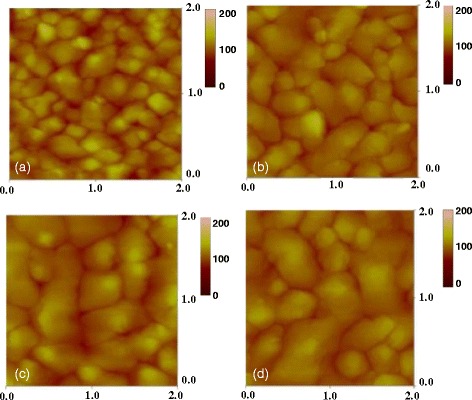
The AFM images of pentacene deposited on **a** IB0, **b** IB10, **c** IB10/PMMA, and **d** IB10/COC dielectrics

The FE-SEM and AFM images show the morphologies of pentacene grown on (a) PB0, (b) PB10, (c) PB10/PMMA, and (d) PB10/COC dielectrics (as shown in Figs. 9 and 10). Pentacene is inherently hydrophobic, and we found that the PB10–COC insulator with the lowest surface energy (47.48 mJ m^−2^) facilitates pentacene growth. The size of pentacene grain domains deposited on the hybrid dielectrics increased as the BaTiO_3_ content was increased in the hybrids. This may be related to the affinity of the dielectric surface for pentacene. The grain size increased as the BaTiO_3_ content was increased from PB0 to PB10. The well-connected domains on the PB10 dielectric provide an efficient channel for charge transport. Inserting the high-dielectric-constant hybrid film induced greater charge carrier densities at the dielectric–semiconductor interface, leading to fulfilling more charge traps at the interface. However, as the BaTiO_3_ content was greater than 12 wt%, the device mobility decreased. This may be attributed to the rougher surface of PB12 with the aggregates of BaTiO_3_ particles, thus interfering with the formation of crystalline structures. After the polymer-modified layer was added, the greater amount of boundary domains restricted charge transport in the semiconductor channel. These results revealed that the hydroxyl-free, nonpolar PMMA and COC materials form a more hydrophobic surface, facilitating crystalline growth. Moreover, the modified layer reduced the traps on the dielectric surface, thereby enhancing the OTFT performance [33–35].

## Conclusions

Pentacene-based OTFTs with a series of high-dielectric-constant IBX hybrid thin films, with different inorganic concentrations and polymer-modified layers used as a dielectric material, were successfully fabricated. The PI–inorganic materials provide a covalent-bonded surface, and the inorganic particles display a high degree of dispersion of BaTiO_3_ nanoparticles in the PI matrix. The dielectric constant of the composites is tunable by changing the concentration of BaTiO_3_ content incorporated with the PI matrix. The device performance and film properties reveal a favorable relationship with the weight percent of BaTiO_3_. Furthermore, the surface morphology and crystallinity of pentacene were significantly improved after the modification of hybrid gate dielectric layers with hydroxyl-free PMMA and COC polymer insulators. These PI hybrid materials demonstrate the development of transparent and environmentally safe gate dielectric materials for applications in transistors and related electronic devices.
